# ONLAY VERSUS RIVES-STOPPA TECHNIQUES IN THE TREATMENT OF INCISIONAL HERNIAS

**DOI:** 10.1590/0102-672020230048e1766

**Published:** 2023-10-13

**Authors:** Alana Kezya Pereira-Rodrigues, João Victor Santos Maceio-Da-Graça, Erik Matheus Lemos de Oliveira Ferreira, Claudio Claudino Alves-Almeida

**Affiliations:** 1 Hospital de Clínicas Gaspar Viana, General Surgery Service – Belém (PA), Brazil

**Keywords:** Hernia, Hernia, abdominal, Seroma, Surgical wound infection, Length of stay, Hérnia, Hérnia abdominal, Seroma, Infecção da ferida cirúrgica, Tempo de internação

## Abstract

**BACKGROUND:**

In the surgical correction of large incisional hernias, the use of a prosthesis is essential in most cases regardless of the technique chosen. The preference is for the polypropylene prosthesis.

**AIMS:**

To compare the onlay and Rives-Stoppa techniques in the correction of incisional hernias, their immediate results, complications, advantages, and disadvantages.

**METHODS:**

Two groups of patients with incisional hernias were analyzed, submitted to the onlay (19 patients) and Rives-Stoppa (17 patients) techniques, and that used polypropylene prostheses. General epidemiological variables, perioperative data variables, and postoperative complications were assessed.

**RESULTS:**

The patients’ epidemiologic profile was similar between both groups. The majority were women (58.4%), with a mean age of 65.5 years and a previous mean body mass index of 41.5 kg/m². The Rives-Stoppa technique was employed in most patients (52.7%). Those submitted to the onlay technique had longer abdominal drainage time and longer hospital stay, as well as a higher incidence of seromas and surgical wound infection.

**CONCLUSIONS:**

The incisional herniorrhaphy technique with the placement of a pre-peritoneal polypropylene mesh by the Rives-Stoppa technique was superior to the onlay due to lower rates of drain use, hospital stay, and postoperative complications.

## RESUMO

**RACIONAL:**

Na correção cirúrgica das grandes hérnias incisionais, independente da técnica escolhida, na maioria das vezes, o uso de uma prótese é imprescindível. A preferência é pelas próteses de polipropileno.

**OBJETIVOS:**

Comparar a técnicas onlay e Rives-Stoppa na correção de hérnias incisionais, os seus resultados imediatos, as complicações, as vantagens e desvantagens.

**MÉTODOS:**

Foram analisados dois grupos de portadores de hérnias incisionais, submetidos à técnica onlay (19 pacientes) e técnica Rives-Stoppa (17 pacientes), utilizando prótese de polipropileno. As variáveis gerais epidemiológicas, variáveis de dados perioperatórios e complicações pós-operatórias foram analisados.

**RESULTADOS:**

O perfil epidemiológico dos pacientes foi semelhante entre os grupos, a maioria eram mulheres (58.4%), com média de idade de 65.5 anos e com índice de massa corpórea (IMC) médio prévio de 41,5kg/m². A técnica de Rives-Stoppa foi utilizada na maioria dos pacientes (52.7%). Pacientes submetidos a técnica onlay apresentaram maior tempo de uso de dreno abdominal e maior tempo de internação hospitalar, além de maior incidência de seromas e infecção de ferida operatória.

**CONCLUSÕES:**

A técnica de herniorrafia incisional com colocação de tela de polipropileno pré-peritoneal, pela técnica de Rives-Stoppa apresentou-se superior à técnica onlay, devido menor taxa de uso de drenos, menor taxa de tempo de internação hospitalar, bem como menor taxa de complicações pós-operatórias.

## INTRODUCTION

For the surgical correction of large incisional hernias, regardless of the technique performed, using a prosthesis is essential in most cases. The preference is for polypropylene prostheses, size 20×30 cm or 30×30 cm, sectioned and adjusted to the size of the abdominal wall defect^12^.

There are four options regarding the location of the prosthesis: premusculoaponeurotic (onlay), retromuscular (underlay or sublay), preperitoneal (underlay or sublay), and intraperitoneal (inlay)^10^.

The onlay or pre-aponeurotic technique consists of positioning the mesh on the anterior aponeurosis of the abdomen, just below the subcutaneous cellular tissue in the pre-fascial region^15^. Among its main advantages, we can highlight the greater technical ease of implementation. Like other techniques, it can be accompanied by myofascial advancement of the abdominal wall, thus corroborating the correction of simple to giant hernias with loss of domain^15^. Another relevant aspect is the easier local approach in situations of superficial surgical site infection, which can be done by simply opening the skin stitches and cleaning the surgical wound. Another advantage is the shorter surgical time^5,15^.

The main disadvantages would be the higher rates of clinically detectable seroma, requiring the use of abdominal drains, which consequently increases the patient's hospitalization period, in addition to higher rates of surgical site infection when compared to the pre-peritoneal technique^6,15^. It is worth mentioning that the close contact of the mesh with the skin in very thin patients or in the areas of reduced subcutaneous cellular tissue, can favor its erosion^15^.

The Rives-Stoppa technique consists of the dissection between the rectus abdominis muscle and the posterior leaflet of its aponeurosis allowing the positioning of an underlay mesh. It is widely accepted in the literature and is the standard recommended in several specialized services for the treatment of abdominal wall hernias due to the non-use of abdominal drains, reduction of morbidity and recurrence rates^4^. Previous prospective observational studies showed a lower recurrence rate in underlay repairs than in onlay repairs^9,11,14^.

Although there is still no evidence in systematic reviews regarding the superiority of any mesh position, the underlay technique is a satisfactory option with positive results as demonstrated in comparative studies^11,13^. Among the disadvantages, it is worth mentioning the higher technical difficulty, longer surgical time, and the need for surgeons’ experience in obeying the technical precepts necessary to obtain better results^1,11^.

The aim of this study was to compare the onlay and Rives-Stoppa techniques in incisional hernia repair, their immediate results, and complications, and discuss the advantages and disadvantages.

## METHODS

The medical records of 36 patients undergoing median incisional herniorrhaphy from 2019 to 2021 were retrospectively analyzed and divided into two groups:

**Group 1**: 19 patients undergoing incisional hernioplasty using the Rives-Stoppa technique.
**Group 2**: 17 patients who underwent incisional hernioplasty using the onlay technique.

Patient information was recorded on a standardized form, including clinical and epidemiological variables, surgical techniques used, and possible complications.

Exclusion criteria were incomplete medical records and patients refusing to participate in the study.

All operations were performed at the Gaspar Vianna Clinical Hospital, in Belém (PA). The study was approved by the Research Ethics Committee under CAAE number 58508522.9.0000.0016.

The Rives-Stoppa technique consisted of identifying the hernia defect, resecting the hernia sac, and fixing the polypropylene mesh in the pre-peritoneal plane. Next, the rectus abdominis aponeurosis was sutured using a continuous suture with 2.0 polypropylene thread, avoiding the contact of the mesh with the subcutaneous tissue.

The onlay technique consisted of identifying the hernia defect, dissecting the anterior leaflet of the rectus abdominis muscle from the subcutaneous tissue, positioning the polypropylene mesh over the anterior aponeurosis of the abdomen, and fixing it with 2.0 polypropylene thread to the aponeurosis.

In order to verify homogeneity among patients, a comparison was performed between the groups considering the following variables: gender, age, comorbidities, smoking, body mass index (BMI), and recurrent hernias.

In addition, the correlation between patients regarding the use of abdominal drain, time of use of the drain, length of hospital stays, and postoperative complications was recorded.

For statistical analysis, a descriptive assessment of clinical and epidemiological profile variables was performed. The chi-square test was employed for categorical variables and the Students *t*-test was used for numerical values. The BioEstat 2.0 program was employed for statistical relevance.

## RESULTS

In this series, the majority of patients were women (58.4%), with a mean age of 65.5 years, and a previous mean BMI of 41.5 kg/m². The Rives-Stoppa technique was employed in most patients (52.7%).

There was no statistically significant difference in age, BMI, comorbidities, and smoking between the two groups analyzed (Table 1).

**Table 1:** General data of the studied cases.

General data					
Variables			Rives-Stoppa technique	Onlay technique	p
Gender (%)					
	Male	15 (41.6)	7 (19.5)	8 (22.1)	0.53
	Female	21 (58.4)	12 (33.4)	9 (25)	
Age (average)		65.5 years			0.15
Comorbities (%)					
	None	9 (25)	6 (16.6)	2 (5.6)	0.12
	One comorbidity	15 (41.6)	7 (19.5)	9 (25)	
	Two comorbidities	9 (25)	4 (11.2)	5 (13.8)	
	More than two comorbidities	4 (11.2)	2 (5.6)	2 (5.6)	
Smoking (%)					
	Yes	19 (52.9)	9 (25)	10 (27.8)	0.49
	No	17 (47.1)	10 (27.8)	7 (19.5)	
BMI (average)		41.55 kg/m²			0.35

BMI: body mass index.

Table 2 shows the perioperative data collected. Patients undergoing incisional hernioplasty using the onlay technique had a greater need for drainage (p=0.0003, p<0.05) and longer drainage time (p=0.0003, p<0.05) than patients undergoing the Rives-Stoppa technique. Hospital stay was longer in the group of patients submitted to the onlay technique compared to the group of patients submitted to the Rives-Stoppa technique (p=0.0038, p<0.05).

**Table 2:** Perioperative data.

Variables		Rives-Stoppa technique (%)	Onlay technique (%)	p
Use of drain				
	Yes	3 (8.3)	13 (36.1)	0.0003
	<7 days	3 (8.3)	10 (27.8)	
	7 to 14 days	0 (0)	3 (8.3)	
	>14 days	0 (0)	0 (0)	
	No use	16 (44.5)	4 (11.2)	
Hospital stay				
	<7 days	14 (38.9)	7 (19.5)	0.0038
	7 to 14 days	2 (5.6)	8 (22.1)	
	>14 days	3 (8.3)	2 (5.6)	

Regarding complications (Table 3), the group submitted to the onlay technique presented higher rates of seroma (p<0.0001, p<0.05) and surgical wound infection (p<0.0001, p<0.05) than the group of patients submitted to the Rives-Stoppa technique.

**Table 3:** Postoperative complications

Postoperative complications				
Variables		Rives-Stoppa technique	Onlay technique	p
Seroma (%)				
	Yes	2 (5.6)	5 (13.9)	<0.0001
	No	17 (47.3)	12 (33.4)	
Surgical wound infection (%)				
	Yes	1 (2.7)	3 (8.3)	<0.0001
	No	18 (50)	14 (38.9)	

## DISCUSSION

This study compared two groups of patients undergoing different surgical techniques for incisional hernia repair.

The patients’ profile was as expected. Most of them were female (58.4% women), the mean age was 65.5 years, and the mean BMI was 41.55 kg/m². In addition, most patients had comorbidities and were smokers, which is consistent with the profile of patients at risk of developing hernias and peri and postoperative complications in general, according to the literature^2^.

The two groups were homogeneous with respect to age, gender, smoking, BMI, and comorbidities. There was no statistically significant difference.

The onlay technique presented higher rates of drainage need (36.1%) (p=0.0003, p<0.05) and incidence of seroma (13.9%) (p<0.0001, p<0.5). According to the literature, these findings are related to the surgical technique itself once it is necessary to create a space between the aponeurosis of the rectus muscle and the subcutaneous tissue to locate the hernial defect and fix the prosthesis properly. And to obtain this space, a dissection using electrocautery is necessary^7^.

Since seroma is an accumulation of fluids between two tissues that were detached, its incidence is also higher due to the use of suction drains for the drainage of these fluids accumulated in the spaces created for placement and fixation of the prosthesis. This large detachment of tissues generates a significant daily volume of liquids in this space, which tends to decrease between five and seven days on average, in most cases^7^.

In the institution where this study was conducted, the drain removal is performed before hospital discharge. For the drain to be safely removed, the volume must be equal to or less than 50 ml within two subsequent days. Thus, it can be observed that in relation to the length of hospital stay, the rate was higher in patients undergoing the onlay technique (27.7%) (p=0.0038, p<0.05).

It was also observed that the rate of surgical wound infection was higher in patients undergoing the onlay technique (p<0.0001, p<0.05). According to the literature, this finding may be correlated with the superficial location of the mesh and the facilitation of bacterial colonization in the area.

The wound complications are a common problem in incisional hernia repair, regardless of the technique used. Some studies show that these complications occur more frequently after onlay repair than in the pre-peritoneal method, although others do not show this correlation^14^.

According to the literature, both seroma and infection are more frequent after the onlay technique due to the greater dissection of the subcutaneous tissue and its contact with the mesh^7^. However, recent meta-analyses comparing onlay and pre-peritoneal repair techniques showed no difference in seroma development, but fewer cases of wound infection were found in the pre-peritoneal group^8^.

Demetrashvili et al.^3^ noted a lower rate of wound complications when comparing retromuscular hernia repair (22.1%) with onlay repair (50.0%) (p<0.001)^8^ which corroborates the results of this study (Table 3). Regarding the seroma rate being higher in the onlay group, even with the use of drain (p=0.00038), this finding corroborates a retrospective study by Hodgson et al. who evaluated the occurrence of postoperative complications after drain placement in various types of hernia repair. They stated that drainage did not decrease the incidence of seroma formation but increased the length of stay of patients^8^. These results were also observed in this study.

## CONCLUSIONS

The incisional herniorrhaphy technique with the placement of a pre-peritoneal polypropylene mesh, using the Rives-Stoppa technique, was superior to the onlay technique due to lower rates of drain use, hospitalization time, and postoperative complications.
